# Human Leukocyte Antigen Class II Alleles Are Associated with Hepatitis C Virus Natural Susceptibility in the Chinese Population

**DOI:** 10.3390/ijms160816792

**Published:** 2015-07-23

**Authors:** Ming Yue, Ke Xu, Meng-Ping Wu, Ya-Ping Han, Peng Huang, Zhi-Hang Peng, Jie Wang, Jing Su, Rong-Bin Yu, Jun Li, Yun Zhang

**Affiliations:** 1 Department of Infectious Diseases, the First Affiliated Hospital of Nanjing Medical University, Nanjing 210029, China; E-Mails: njym08@163.com (M.Y.); asian_peace@126.com (Y.-P.H.); 2 Department of Acute Infection Diseases, Jiangsu Province Center for Disease Prevention and Control, Nanjing 210009, China; E-Mail: xuke923@163.com; 3 Department of Epidemiology and Biostatistics, School of Public Health, Nanjing Medical University, Nanjing 211166, China; E-Mails: wumengping1990@163.com (M.-P.W.); hp19880310@163.com (P.H.); zhihangpeng@njmu.edu.cn (Z.-H.P.); sujing@njmu.edu.cn (J.S.); rongbinyu@njmu.edu.cn (R.-B.Y.); 4 School of Nursing, Nanjing Medical University, Nanjing 211166, China; E-Mail: wangjie.nj@gmail.com; 5 Institute of Epidemiology and Microbiology, Huadong Research Institute for Medicine and Biotechnics, Nanjing 210002, China

**Keywords:** human leukocyte antigen, hepatitis C virus, infection, polymorphism, outcome

## Abstract

Human leukocyte antigen (HLA) class II molecule influences host antigen presentation and anti-viral immune response. The aim of this study was to investigate whether single nucleotide polymorphisms (SNPs) within HLA class II gene were associated with different clinical outcomes of hepatitis C virus (HCV) infection. Three HLA class II SNPs (rs3077, rs2395309 and rs2856718) were genotyped by TaqMan assay among Chinese population, including 350 persistent HCV infection patients, 194 spontaneous viral clearance subjects and 973 HCV-uninfected control subjects. After logistic regression analysis, the results indicated that the rs2856718 TC genotype was significantly associated with the protective effect of the HCV natural susceptibility (adjusted OR: 0.712, 95% CI: 0.554–0.914) when compared with reference TT genotype, and this remained significant after false discovery rate (FDR) correction (*p* = 0.024). Moreover, the protective effect of rs2856718 was observed in dominant genetic models (adjusted OR: 0.726, 95% CI: 0.574–0.920), and this remained significant after FDR correction (*p* = 0.024). In stratified analysis, a significant decreased risk was found in rs2856718C allele in the male subgroup (adjusted OR: 0.778, 95% CI: 0.627–0.966) and hemodialysis subgroup (adjusted OR: 0.713, 95% CI: 0.552–0.921). Our results indicated that the genetic variations of rs2856718 within the HLA-DQ gene are associated with the natural susceptibility to HCV infection among the Chinese population.

## 1. Introduction

Hepatitis C virus (HCV) is estimated to infect 185 million people worldwide, which poses an increasingly severe global health burden [1]. The prevalence rate of HCV infection is about 3.2% in the Chinese general population [1]. The outcomes of HCV infection varied widely. Only approximately 20%–30% of cases with acute infection can result in spontaneous asymptomatic clearance, while the remaining cases will develop chronic infection that may lead to cirrhosis and hepatocellular carcinoma (HCC) [1]. The pathogenetic mechanisms involved in the HCV infection are not completely elucidated. It is well-known that the pathogen and host factors, including viral genotype, viral load, host gender, age, ethnicity, the immune response, the metabolic co-factors [2], and, especially, host genetic background, influence the progression of HCV infection.

Genes encoding human histocompatibility leukocyte antigens (HLA) molecules (also known as the human major histocompatibility complex), are divided into three subregions, and HLA classes I, II, and III, are located on the short arm of chromosome 6 [3]. HLA class I and II genes are found to be highly polymorphic in the human genome, 40% of whom are strongly associated with the human immune function [4]. It has been generally accepted that the specific cellular immune response plays a central role in the outcome of HCV infection, and the effective immune struggle against the invading virus is harmonized by strong CD4+ and CD8+ T cell responses through recognizing HCV-derived antigens presented by the HLA class II and class I molecules, respectively [5].

HLA class II molecules are expressed on antigen presenting cells (APCs), including dendritic cells (DCs), B cells, macrophages, thymic epithelial cells and activated T cells. The recognition of presented exogenous antigen peptide to CD4+ T cells leads to the secretion of cytokines and the differentiation of T cells [6]. There are three major class II molecules, namely, HLA-DP, -DQ, and -DR. Each HLA class II molecules are heterodimers composed of two polypeptides chains, α and β chain, which has four domains: the highly polymorphic α1 and β1 domains (act as the antigen-binding site), and the highly conserved α2 and β2 domains (act as the immunoglobulin-like domains) [7]. Therefore, it is conceivable that some mutations on certain site of HLA class II gene may influence the capability of antigen presentation and the production of cytokines, thus affect the host immune response and the progression HCV infection.

To date, increasing evidence revealed that a multitude of single nucleotide polymorphisms (SNPs) located within the HLA class II genes are related to the progression and outcomes of HCV infection, including HCV natural susceptibility and clearance. A meta-analysis reported that HLA DQB1*0301, DRB1*1101 alleles are protective alleles for spontaneous clearance of HCV infection worldwide [8], and a conventional genotyping study showed that HLA-DQA1*0501, DQB1*0301, DQB1*0201 and DRB1*0401 alleles influence the serum alanine transaminase (ALT) level of chronic HCV infection in the Chinese population [9]. HLA DR4, DQB1*0401/0402/0503, DRB1*0405 are associated with viral persistence and chronic liver disease in Japanese [10,11,12]. Besides, many HLA class II alleles were considered to correlate with the natural susceptibility, viral persistence/clearance, liver cirrhosis, progressive liver damage, viral load, response to interferon therapy, vertical transmission, and extra-hepatic manifestations of HCV infection in global populations [13]. A recent genome-wide association study (GWAS) demonstrated that HLA class II and IL-28B are independently associated with spontaneous resolution of HCV infection in individuals of European and African ancestry [14]. Additionally, many HLA class II alleles were considered as candidate genetic markers in hepatitis B virus (HBV) infection, response to HBV vaccination and interferon therapy worldwide [13,15].

It is currently not known whether HLA-DP: rs3077, rs2395309 and HLA-DQ: rs2856718, which are frequently reported to be related to HBV infection among Japanese, Korean and many other ethnic groups, also have an association with the outcomes of HCV infection among Chinese population. In light of the potential influence of HLA class II polymorphisms on the immune response in HCV infection, we examined the relationships of the three SNPs and HCV infection outcomes among the Chinese Han population. We found the genetic variations of rs2856718 are associated with the natural susceptibility to HCV infection in the Chinese population.

## 2. Results

### 2.1. Basic Characteristics

All subjects’ characteristics are shown in Table 1. Group A consisted of 370 cases (204 males, 166 females; mean age 46.86 ± 13.05 years). Group B included 194 individuals (110 males, 84 females; mean age 47.03 ± 11.26 years). Group C included 973 subjects (531 males, 442 females; mean age 48.42 ± 13.72 years). The recorded age refers to the age at study enrollment. No significant age and gender difference was observed among the three groups (*p* > 0.05). However, fewer hemodialysis (HD) patients were found in Group A (HCV persistent carriers) than Group B (HCV natural clearance subjects) or Group C (healthy persons) (*p* < 0.001 for both comparisons). Additionally, the differences of ALT level, aspartate transaminase (AST) level, the route of infection and viral genotype among the three groups were statistically significant (all *p* < 0.001).

**Table 1:** Demographic and clinical characteristics of Hepatitis C virus (HCV) persistent infection, spontaneous clearance and healthy control populations.

Variables	Group A (%)	Group B (%)	Group C (%)	*p*
	*n* = 370	*n* = 194	*n* = 973	
Age (mean ± SD)	46.86 ± 13.05	47.03 ± 11.26	48.42 ± 13.72	0.10 ^a,#^
Gender				0.86 ^b,#^
Male	204 (55.1)	110 (56.7)	531 (54.6)	
Female	166 (44.9)	84 (43.3)	442 (45.4)	
ALT (median (IQR), U/L)	23.00 (34.00)	19.00 (20.50)	9.00 (9.00)	<0.001 ^c,**†**^
AST (median (IQR), U/L)	30.00 (27.00)	24.00 (15.50)	16.00 (12.00)	<0.001 ^c,**†**^
Routes of infection				<0.001 ^b,**†**^
Drug use	155 (41.9)	36 (18.6)	246 (25.3)	
Hemodialysis	74 (20.0)	89 (45.9)	551 (56.6)	
Paid blood donation	141 (38.1)	69 (35.6)	176 (18.1)	
HCV genotypes				<0.001 ^b,**†**^
1	236 (63.8)	114 (58.8)	–	
Non-1	51 (13.8)	53 (27.3)	–	
Mixed	83 (22.4)	27 (13.9)	–	

Group A: HCV persistent carriers; Group B: HCV natural clearance subjects; Group C: Healthy controls, ALT: alanine transaminase; AST: aspartate transaminase; SD: standard deviation; IQR: interquartile range; Non-1: genotype 2, 3 and unknown; Mixed: co-infected with genotype 1, 2 and 3. ^a^ *p* value of One-Way ANOVA among three groups; ^b^ *p* value of χ^2^-test among three/two groups; ^c^ *p* value of Kruskal-Wallis test or Mann-Whitney *U* test among three/two groups; ^#^ All *p* > 0.05, between any two groups; **^†^** All *p* < 0.001, between any two groups.

The allele distributions for rs3077, rs2395309 and rs2856718 of Group B and Group C, which were considered as the control groups in the different comparisons, were in Hardy-Weinberg Equilibrium (HWE) expectations (Group B: *p* = 0.729 for rs3077, *p* = 0.852 for rs2395309, *p* = 0.413 for rs2856718; Group C: *p* = 0.863 for rs3077, *p* = 0.694 for rs2395309, *p* = 0.096 for rs2856718). Linkage disequilibrium (LD) information about HLA class II variations is shown in Supplementary Table 2 (Table S2).

### 2.2. Association Analysis of HLA Class II Gene Polymorphisms and Haplotype with Susceptibility and the Resolution of HCV Infection

The genotype frequencies of three SNPs between cases (both clearance and carriers) and controls were examined. When compared with the healthy control group (Group C), logistic regression analyses showed that the carriage of rs2856718 TC genotype was associated with a decreased risk of the susceptibility to HCV (adjusted OR: 0.712, 95% CI: 0.554–0.914; *p* = 0.008) in HCV-infected persons when compared with reference TT genotype, and this remained significant after accounting for multiple comparisons (*p* = 0.024). Moreover, the benefit genotypic effect of rs2856718 was observed in dominant genetic models (adjusted OR: 0.726, 95% CI: 0.574–0.920; *p* = 0.008), and this remained significant after accounting for multiple comparisons (*p* = 0.024) (Table 2). In additive genetic model, no significant effect of rs2856718 variant genotypes on the HCV infection risk was found (adjusted OR: 0.856, 95% CI: 0.733–1.000; *p* = 0.050). However, no significant association was observed between rs3077 and rs2395309 with HCV susceptibility (Table 2).

To decrease the bias of sex, age, route of infection, and viral genotype in population sampling, we further conducted the stratified analysis. The results of rs2856718 indicated that, compared with the TT genotype, a significant decreased risk was found in C allele in the male subgroup (adjusted OR: 0.778, 95% CI: 0.627–0.966; *p* = 0.023), which is stratified according the previous literature [16], and HD subgroup (adjusted OR: 0.713, 95% CI: 0.552–0.921; *p* = 0.010) (Table 3). Nevertheless, no significant effect of rs3077 or rs2395309 on the susceptibility or the resolution of HCV infection risk was found in different strata (all *p* > 0.05).

**Table 2:** Distributions of HLA class II genotypes among persistent infection, spontaneous clearance and control group.

SNPs (Genotype)		Group A	Group B	Group C	OR (95% CI) ^a^	*p* ^a^/*p* ^a′^	OR (95% CI) ^b^	*p* ^b^/*p* ^b′^
		*n* (%)	*n* (%)	*n* (%)	The Outcome: HCV Susceptibility		The Outcome: HCV Clearance	
rs3077								
	CC	143 (38.8)	76 (39.4)	406 (41.9)	1.00	–	1.00	–
	TC	173 (46.9)	92 (47.7)	445 (45.9)	1.117 (0.888–1.406)	0.345/0.345	0.964 (0.648–1.432)	0.855/0.855
	TT	53 (14.4)	25 (13.0)	119 (12.3)	1.271 (0.906–1.783)	0.165/0.165	1.227 (0.687–2.191)	0.490/0.706
Additive model					1.125 (0.961–1.316)	0.143/0.143	1.068 (0.817–1.396)	0.631/0.826
Dominant model					1.149 (0.924–1.429)	0.211/0.211	1.018 (0.700–1.482)	0.924/0.956
rs2395309								
	GG	140 (37.8)	74 (38.3)	401 (41.3)	1.00	–	1.00	–
	AG	176 (47.6)	92 (47.7)	451 (46.4)	1.118 (0.888–1.408)	0.342/0.345	0.953 (0.640–1.420)	0.814/0.855
	AA	54 (14.6)	27 (14.0)	120 (12.3)	1.312 (0.937–1.835)	0.113/0.165	1.116 (0.631–1.973)	0.706/0.706
Additive model					1.138 (0.973–1.332)	0.106/0.143	1.030 (0.789–1.345)	0.826/0.826
Dominant model					1.158 (0.931–1.441)	0.187/0.211	0.989 (0.679–1.442)	0.956/0.956
rs2856718								
	TT	108 (29.8)	69 (36.1)	255 (26.7)	1.00	–	1.00	–
	TC	179 (49.4)	87 (45.5)	502 (52.5)	**0.712 (0.554–0.914)**	**0.008/0.024**	1.094 (0.718–1.667)	0.676/0.855
	CC	75 (20.7)	35 (18.3)	199 (20.8)	0.763 (0.559–1.040)	0.087/0.165	1.177 (0.692–2.000)	0.548/0.706
Additive model					0.856 (0.733–1.000)	0.050/0.143	1.086 (0.837–1.410)	0.536/0.826
Dominant model					**0.726 (0.574–0.920)**	**0.008/0.024**	1.118 (0.753–1.659)	0.581/0.956

SNPs: single nucleotide polymorphisms, Group A: HCV persistent carriers; Group B: HCV natural clearance subjects; Group C: Healthy controls; Group (A + B): HCV-infected patients; ^a^ The *p* value, odds ratio (OR), 95% confidence intervals (CI) of Group (A + B) *versus* Group C were calculated on the basis of the logistic regression model, adjusted by gender, age and route of infection; ^b^ The *p* value, odds ratio (OR), 95% confidence intervals (CI) of Group A *versus* Group B were calculated on the basis of the logistic regression model, adjusted by gender, age, route of infection, and viral genotype. ^a′,b′^ Multiple testing: using False Discovery Rate. Bold type indicates statistically significant results.

**Table 3:** Stratified analysis of HLA rs2856718 among persistent infection, spontaneous clearance and control group.

Supgroups (Genotypes)	Group A *n* (%)			Group B *n* (%)			Group C *n* (%)			OR (95% CI) ^a^	*p* ^a′^	OR (95% CI) ^b^	*p* ^b^
	TT	TC	CC	TT	TC	CC	TT	TC	CC	The outcome: HCV susceptibility		The outcome: HCV clearance	
Age													
≤40	37 (27.6)	75 (56.0)	22 (16.4)	20 (30.8)	30 (46.2)	15 (23.1)	69 (24.0)	153 (53.1)	66 (22.9)	0.886 (0.670–1.171)	0.393	0.943 (0.604–1.474)	0.798
>40	71 (31.1)	104 (45.6)	53 (23.2)	49 (38.9)	57 (45.2)	20 (15.9)	186 (27.8)	349 (52.2)	133 (19.9)	0.858 (0.710–1.037)	0.112	1.188 (0.859–1.644)	0.297
Gender													
Male	64 (31.8)	102 (50.7)	35 (17.4)	39 (35.8)	49 (45.0)	21 (19.3)	135 (25.9)	286 (54.8)	101 (19.3)	**0.778 (0.627–0.966)**	**0.023**	0.958 (0.678–1.354)	0.807
Female	44 (27.3)	77 (47.8)	40 (24.8)	30 (36.6)	38 (46.3)	14 (17.1)	120 (27.6)	216 (49.8)	98 (22.6)	0.945 (0.754–1.184)	0.621	1.295 (0.860–1.950)	0.216
Routes of infection													
Drug use	36 (24.3)	76 (51.4)	36 (24.3)	12 (35.3)	15 (44.1)	7 (20.6)	62 (25.9)	125 (52.3)	52 (21.8)	1.021 (0.773–1.348)	0.883	1.363 (0.792–2.347)	0.264
Hemodialysis	32 (43.8)	26 (35.6)	15 (20.5)	35 (39.8)	37 (42.0)	16 (18.2)	144 (26.5)	283 (52.1)	116 (21.4)	**0.713 (0.552–0.921)**	**0.010**	1.046 (0.682–1.605)	0.836
PDD	40 (28.4)	77 (54.6)	24 (17.0)	22 (31.9)	35 (50.7)	12 (17.4)	49 (28.2)	94 (54.0)	31 (17.8)	0.988 (0.726–1.343)	0.937	1.031 (0.657–1.620)	0.894
HCV genotypes													
1	69 (29.9)	120 (51.9)	42 (18.2)	45 (40.2)	49 (43.8)	18 (16.1)	–	–	–	–	–	1.192 (0.847–1.678)	0.314
Non-1	14 (27.5)	22 (43.1)	15 (29.4)	13 (25.0)	25 (48.1)	14 (26.9)	–	–	–	–	–	0.980 (0.558–1.721)	0.943
Mixed	25 (31.3)	37 (46.3)	18 (22.5)	11 (40.7)	13 (48.1)	3 (11.1)	–	–	–	–	–	1.292 (0.656–2.545)	0.459

Group A: HCV persistent carriers; Group B: HCV natural clearance subjects; Group C: Healthy controls; Group (A + B): HCV-infected patients. Non-1: genotype 2, 3 and unknown; Mixed: co-infected with genotype 1, 2 and 3; HD: hemodialysis; PDD: paid blood donation. ^a^ The *p* value, odds ratio (OR), 95% confidence intervals (CI) of Group (A + B) *versus* Group C were calculated on the basis of the logistic regression model, adjusted by gender, age, route of infection, and/or viral genotype; ^b^ The *p* value, odds ratio (OR), 95% confidence intervals (CI) of Group A *versus* Group B were calculated on the basis of the logistic regression model, adjusted by gender, age, route of infection, and/or viral genotype. Bold type indicates statistically significant results.

The three-locus haplotypes were consisted of rs3077, rs2395309 and rs2856718 variant alleles. No significant difference was observed between any haplotype and the susceptibility or the resolution of HCV infection after logistic regression analyses (all *p* > 0.05) (Table S3).

## 3. Discussions

Evidence is accruing that HLA class II molecules are associated with the outcomes of hepatitis B and C. In this study, we firstly explored the associations of rs3077 (C>T), rs2395309 (G>A), and rs2856718 (T>C), with the susceptibility and the resolution of HCV infection among Chinese Han population, and found that rs2856718C allele carriers had a low risk for the natural susceptibility to HCV infection both in the overall SNP analysis and some subgroups of stratified analysis after the adjustment for gender, age, and route of infection.

The cell surface glycopeptides of the HLA-DP and DQ belong to HLA Class II molecules, which are strongly involved in the presentation of HCV specific epitopes to CD4+ T cells [17]. It has been mainly described that the patients with established chronic HCV infection frequently have much weaker CD4+ T-cell responses specific to HCV antigens than those with resolved infection [5]. The three SNPs are located in human chromosome 6p21.3 region, without defined functions. The rs3077 (chromosome position: 33141000) is found within 3′-UTR region of HLA-DPA1 gene, with rs2395309 (chromosome position: 33134224) nearby. Neither of them has the direct protein-coding function. In addition, rs3077 and rs2395309 are in tight linkage disequilibrium (Table S2). The bioinformatics analyses using the SNP function prediction website (http://snpinfo.niehs.nih.gov/) indicated that rs3077 might be a transcription factor binding site (TFBS) of microRNA, including human miR-1244, miR-199, miR-140, miR-616, *etc.* Thus the polymorphisms of rs3077 may influence the binding affinity to these microRNAs, which are correlated well with the post-transcriptional regulation [18]. As many previous studies showed, the major allele (G) of rs3077 is closely related with the higher risk of chronic hepatitis B in Chinese [19,20,21,22,23], Japanese, Korean and Thai [24,25], as well as the major allele (G) of rs2395309 does in Chinese [20,26]. Besides, an *in vitro* functional research revealed that rs3077G alleles was associated with the lower expression of messenger RNA expression of HLA-DPA1 in liver tissues derived from the chronic hepatitis B patients of non-Hispanic European ancestry, which may be related to the increased risk of chronic HBV infection [27]. And rs3077 was also found to influence the methylation level of HLA-DPB1 in adult cerebellum samples [28].

Although many studies have indicated the role of rs3077 and rs2395309 in HBV infection, in the present study, no association was found between rs3077 or rs2395309 polymorphisms in either the susceptibility or the resolution of HCV infection, which were not in agreement with the previous studies. The most important reason for these differences is that, although both HBV and HCV are hepatotrophic, and likely to result in liver diseases, the molecular virology and specific immune responses of the two viruses differ profoundly. After reviewing the previous literature, we found that the influence of some genetic variants (of HLA class II gene) on HBV infection is almost the same as HCV infection, but some are totally different. For example, HLA DR*13 was reported to confer a consistent benefit against the vertical transmission of HBV and HCV among Chinese and Italian populations [29,30,31]. However, HLA DRB1*11 and HLA DQB1*0301 play opposite roles in the course of the two viral infections [13]: they exert protective effect against HCV persistence, but were considered as risk factors for persistent HBV infection. Beyond that, the functional study on rs3077 [27] was an *in vitro* assay conducted in the liver tissues of the European chronic hepatitis B patients, so the findings may not completely represent the associations of rs3077 with HCV infection among other ethnic groups. Additional functional studies are needed to verify the relationship between the genetic variants of 3077 with HCV infection across ethnic population groups.

Rs2856718 is located in the intergenic region between HLA-DQB1 and HLA-DQA2. Its exact role in gene expression is not clear, and no study on the associations between the SNP rs2856718 and HCV infection has been published. A previous genome-wide association study (GWAS) indicated that the rs2856718G allele had an independently increased risk for chronic hepatitis B (CHB) susceptibility in a Japanese population [32], while another study found this allele was associated with the reduced risk of Saudi Arabian patients to progress into chronic HBV infection [33]. The different results found in the above studies on HBV infection could be due to the difference reference population and the more or less racial differences, especially in the extremely complex regulation of HLA class II expression. It is worth noting that two Chinese research teams confirmed that rs2856718G was strongly related to the decreased risk of chronic HBV infection and host hepatocellular carcinoma (HCC) in Chinese Han patients [23,34], which was somewhat similar with our observations of the protective effect of rs2856718C (equivalent to rs2856718G) allele on HCV infection in the Chinese population.

In the stratified analyses of rs2856718 adjusted by logistic regression, it is revealed that rs2856718C allele also confers a protective effect against HCV susceptibility in male and HD subgroups. The HD patients always received the higher HCV inoculum than drug users and paid blood donors, which may contribute to the inadequate HCV-specific immune response in HD patients. Additionally, epidemiologic evidence has suggested that sex differences exist in host immune responses to HCV infection. The HCV infection rate appears to be higher in men than in women. Since the possible estrogen's protection from HCV infection was insufficient in men, and the HCV-specific immune response may be inadequate in HD patients, the male and HD rs2856718C carriers were more prone to mainly gain the genetic benefit, which was also related to the relative strength of the influence of genetic factor, gender, and routes of infection in HCV infection. Further studies are warranted to confirm the association between rs2856718 and HCV infection in other ethnicities and explore the functional contribution of rs2856718C in the immune response to HCV infection.

The possible deficiencies of this study are the difficulty of learning the exact age and infective dose of HCV infection, and this may affect the host immune response and the outcomes of HCV infection. Besides, to reduce the selection bias in this study, the confounding factors were matched or adjusted with the logistic regression. However, more large survey samples are needed to confirm the findings.

## 4. Experimental Section

### 4.1. Ethic Statement

The study was conducted according to the Declaration of Helsinki (10th revision, 2013) [35], and the research protocol was approved by the institutional review board of Huadong Research Institute for Medicine and Biotechnics (Project identification code: 81072343, Date: 16 March 2006). The written informed consent of all participants were acquired.

### 4.2. Subjects

A total of 1537 individuals were consecutively recruited for the analysis of genotype distribution, including 437 addicts enrolled from Nanjing compulsory drug rehabilitation center (Nanjing, China) from May 2006 to December 2009, 714 hemodialysis (HD) patients enrolled from nine hospital hemodialysis centers in southern China from November 2008 to December 2009, and 386 paid blood donors enrolled from Danyang (China) in April 2011, respectively.

All subjects were divided into three groups as follows: (1) Group A: the cases of chronic HCV infection contained 370 persons (155 addicts, 74 HD patients and 141 paid blood donors) with anti-HCV antibodies and HCV-RNA in sera; (2) Group B: the cases of spontaneous viral clearance contained 194 persons (36 addicts, 89 HD patients and 69 paid blood donors) with anti-HCV antibodies and without HCV-RNA in sera; and (3) Group C: the HCV-uninfected controls contained 973 persons (246 addicts, 551 HD patients and 176 paid blood donors) without anti-HCV antibodies or HCV-RNA in sera. Group C were matched to Group A or Group B for age (5-year interval), sex and geographic location (town and country). Subjects co-infected with any other hepatotropic virus or human immunodeficiency virus (HIV), or treated with antiviral drug, or with any evidence of other liver diseases (autoimmune hepatitis, metabolic disorder, alcohol liver and drugs-induced liver injury) during the study were excluded. All serological tests were conducted for validation at least three times separately within six consecutive months during the follow-up. All patients were diagnosed by experienced physicians on the basis of clinical interviews, laboratory findings and international criteria [36].

Structural interview and standardized questionnaires were used for collecting the information of the possible risk factors involved in the infection of HCV, as previously reported in literature [37], including age, gender, geographic areas, race, history of HCV infection, environmental risk exposure histories of HCV acquisition and so on. The route of infection was determined on basis of the subjects’ exposure history and high-risk behavior acquired in the interview or from the questionnaires. To collect the sufficient reliability data in the study, a quality assurance scheme for epidemiological investigation was established and implemented strictly. The demographic and clinical characteristics of the subjects are presented in Table 1.

### 4.3. HCV Serological Test and Genotyping

All subjects’ blood samples (5 mL) were collected in EDTA tubes and centrifuged to isolate plasma, which then were stored at −20 °C until further processing. HCV-specific antibodies were determined using the third generation enzyme-linked immunosorbent assay (ELISA) (Architect Anti-HCV assay; Abbott Laboratories, Abbott Park, IL, USA). HCV RNA was quantified in plasma samples using commercially available kits (Cobas TaqMan HCV Test, Roche Diagnostics, Mannheim, Germany) according to the manufacturer’s recommendations. HCV genotyping was performed by detecting the viral genotype-specific antibodies using the Murex HCV Serotyping 1–6 Assay ELISA Kit (Abbott, Wiesbaden, Germany) [38].

### 4.4. HLA SNPs Selection and Genotyping

On basis of the literature reports about HLA class II SNPs associated with HCV or HBV infection worldwide, especially in Asian populations, three SNPs were selected as candidates, including HLA-DP: rs3077, rs2395309, and HLA-DQ: rs2856718, with the criteria of minor allele frequency (MAF) >5% in the database of CHB population of HapMap (http://www.hapmap.org).

Genomic DNA was extracted from peripheral leukocytes using the traditional proteinase K and phenol-chloroform method. Rs3077, rs2395309 and rs2856718 were genotyped on the Applied Biosystems 7900 System (Applied Biosystems, Foster City, CA, USA), and analyzed using the TaqMan allelic discrimination assay (Sequence Detection System software, version 2.3; Applied Biosystems, Foster City, CA, USA). The information of specific TaqMan probes, forward and reverse primers are shown in the supplemental Table 1 (Table S1). The operators were blinded to the subjects’ clinical data. In repeated experiments conducted on a 10% of samples randomly selected, a 100% consistency rate was yielded. The success rate of genotyping was above 96%.

### 4.5. Statistical Analysis

Data was input into a database using EpiData 3.1 with dual-entry verification by two operators. Quantitative variables were presented as mean ± standard deviations (SD). The differences of the subjects’ demographic and clinical characteristics, and the distributions of HLA allele frequencies among groups were compared using χ^2^ test, Student’s *t*-test, Student-Newman-Keuls test or one-way analysis of variance (ANOVA) where applicable. Testing for deviations from Hardy-Weinberg equilibrium (HWE) was assessed by a goodness-of-fit χ^2^ test. Linkage disequilibrium (LD) (parameter *r*^2^ and D′) was determined using Haploview software (version 4.2, Massachusetts Institute of Technology, Cambridge, MA, USA). Haplotypes were estimated based on the observed genotypes using the PHASE software (version 2.1, University of Chicago, Chicago, IL, USA). Binary logistic regression analysis was applied with adjustment for confounding factors (age, gender, HCV genotype and route of infection) to estimate the associations of HLA SNPs with natural susceptibility and chronicity risks in HCV infection by computing the odds ratios (ORs) and their 95% confidence intervals (CIs). The false discovery rate approach (FDR), which is less conservative than the ordinary Bonferroni correction, was applied to correct for multiple comparisons. All calculations were performed using SPSS software (version 17.0; SPSS Institute, Chicago, IL, USA) and SAS software (version 9.1.3; SAS Institute, Cary, NC, USA). A two-sided *p* value of less than 0.05 was considered statistically significant.

## 5. Conclusions

Overall, the results of this study suggest that HLA class II SNP rs2856718 is associated with susceptibility to HCV infection in the Chinese population. This genetic variant might serve as a potential biomarker in the progression of HCV infection.

## Supplementary Materials

Supplementary materials can be found at http://www.mdpi.com/1422-0067/16/08/16792/s1.

## Abbreviations

HCV: hepatitis C virus
CHC: chronic hepatitis C
HCC: hepatocellular carcinoma
HLA: human leukocyte antigen
MHC: major histocompatibility complex
Th1 (2, 17, 19): helper T cell 1 (2, 17, 19)
SVR: sustained virological response
IFN-α: interferon-alpha
SNP: single nucleotide polymorphism
HD: hemodialysis
MAF: minor allele frequency
ELISA: enzyme-linked immunosorbent assay
HWE: Hardy-Weinberg equilibrium
LD: linkage disequilibrium
ANOVA: analysis of variance
OR: odds ratio
CI: confidence interval
SD: standard deviations
ALT: alanine transaminase
UTR: untranslated region
GWAS: genome-wide association study
TFBS: transcription factor binding site
