# Supplementary material for: Human Leukocyte Antigen Class II Alleles Are Associated with Hepatitis C Virus Natural Susceptibility in the Chinese Population
Source: Int J Mol Sci. 2015 Jul 23;16(8):16792–805. doi: 10.3390/ijms160816792 (PMC4581170; doi:10.3390/ijms160816792)
Supplement: Supplementary file 1 [file ijms-16-16792-s001.pdf]

Supplementary Information

Table S1. Information of primers and probes for TaqMan allelic discrimination.

SNPs (Genotype)	Nearby Gene	Region	MAF [a/b]	TaqMan-MGB Probe/Primers Sequences (5′–3′)
rs3077 C>T	HLA-DPA1	3-UTR	0.352/0.378	Probe-C: FAM-AAACTACCCCAGTGGC-MGB<br>Probe-T: HEX-AAACTACTCCAGTGGCT-MGB<br>Forward primer: TCAGCTTTTCTTCTCACTTCATGTG<br>Reverse primer: GAGCTTGAAGGGTCAGCAATTC
rs2395309 G>A	HLA-DOA‖HLA-DPA1	N/A	0.355/0.378	Probe-G: FAM-CAGATAGTTGTAAGTGAGATG-MGB<br>Probe-A: HEX-CAGATAGTTATAAGTGAGATGG-MGB<br>Forward primer: GGTCTTATCCAGTTGGTGGTCAA<br>Reverse primer: AGAGGGCTATGGGAAGAGAATGA
rs2856718 T>C	HLA-DQB1‖HLA-DQA2	Intergenic	0.471/0.475	Probe-T: FAM-AGAGCTGTTTAATCC-MGB<br>Probe-C: HEX-AGAGCTGTCTAATCC-MGB<br>Forward primer: GAGCTCCCTCTGGCAGGTT<br>Reverse primer: TATGCTTTCACCAACTTCCTTCAC

MAF: minor allele frequency. [a] minor allele frequencies in control group; [b] minor allele frequencies from HapMap of Han Chinese in Beijing, China (CHB) (dbSNP, build128; available at http://www.ncbi.nlm.nih.gov/SNP/).

Table S2. Linkage disequilibrium information of HLA class II single nucleotide polymorphisms.

	Group A and Group B [a]			Group (A + B) and Group C [b]		
	**Rs3077**	**Rs2395309**	**Rs2856718**	**Rs3077**	**Rs2395309**	**Rs2856718**
Rs3077	–	1.0 [c]	0.194 [c]	–	0.993 [c]	0.16 [c]
Rs2395309	0.957 [d]	–	0.202 [c]	0.971 [d]	–	0.159 [c]
Rs2856718	0.031 [d]	0.036 [d]	–	0.016 [d]	0.016 [d]	–

Group A: HCV persistent female carriers; Group B: HCV natural clearance female subjects; Group C: Healthy female controls; Group (A + B): HCV-infected female patients. [a] Linkage disequilibrium information of the comparison between "Group A and Group B" with "Group B" served as a control group; [b] Linkage disequilibrium information of the comparison between "Group (A + B) and Group C" with "Group C" served as a control group; [c] D′ value; [d] R^2 value.

Table S3. Haplotypes frequencies constituted with polymorphisms of HLA class II SNPs among three groups.

**Haplotype [a]**	**Group A (%)**	**Group B (%)**	**Group C (%)**	**OR (95% CI) [b]**	***p* [b]**	**OR (95% CI) [c]**	***p* [c]**
	***n* = 740**	***n* = 388**	***n* = 1946**	**The Outcome: HCV Susceptibility**		**The Outcome: HCV Clearance**	
CGT	301 (40.7)	173 (44.6)	809 (41.6)	1	1	1	1
TAC	181 (24.5)	89 (22.9)	470 (24.1)	0.975 (0.804–1.182)	0.794	1.094 (0.785–1.525)	0.597
CGC	154 (20.8)	69 (17.8)	442 (22.7)	0.840 (0.687–1.029)	0.092	1.194 (0.835–1.707)	0.332
TAT	99 (13.4)	53 (13.7)	212 (10.9)	1.277 (1.001–1.630)	0.050	1.190 (0.794–1.782)	0.399
Others [d]	5 (0.6)	4 (1.0)	13 (0.7)	0.904 (0.377–2.169)	0.821	0.417 (0.108–1.611)	0.205

SNPs: single nucleotide polymorphisms, Group A: HCV persistent carriers; Group B: HCV natural clearance subjects; Group C: Healthy controls; Group (A + B): HCV-infected patients; [a] Order of single nucleotide polymorphisms comprising the HLA class II haplotypes: rs3077, rs2395309 and rs2856718; [b] Logistic regression model, adjusted by age, and route of infection for Group (A + B) *versus* Group C; [c] Logistic regression model, adjusted by gender, age, route of infection, and viral genotype for Group A *versus* Group B; [d] Haplotypes with a frequency of less than 5% were combined with others.
